# Effect of ^60^Co γ-rays on dried figs adsorption isotherms and thermodynamic properties

**DOI:** 10.3389/fnut.2022.940111

**Published:** 2022-10-11

**Authors:** Ahmed Irchad, Rachid Razouk, Rachida Ouaabou, Mohamed Mouhib, Lahcen Hssaini

**Affiliations:** ^1^Faculty of Sciences and Technics, University of Comoros, Moroni, Comoros; ^2^National Institute of Agricultural Research (INRA), Rabat, Morocco; ^3^Faculty of Sciences Semlalia, Cadi Ayyad University, Marrakesh, Morocco

**Keywords:** gamma irradiation, adsorption isotherms, thermodynamic properties, post-harvest storage, Cobalt radioisotopic source, *Ficus carica* L.

## Abstract

Irradiation is one of the promising food preservation techniques, but few are known about its impact on foods' water vapor change. In this research, the impact of gamma irradiation on moisture adsorption isotherms of dried figs, one of the most emblematic foods of the Mediterranean diet, at increasing doses (0, 1, 1.5, and 2 kGy) was investigated. The isotherms data of equilibrium points displayed a sigmoid-shaped curve of the type II pattern for both controlled and irradiated dried figs, with a notable effect of irradiation on equilibrium moisture content, which revealed a decreasing pattern as irradiation dose and temperature increase. This effect was also seen in data fitting, where GAB model showed the best prediction statistics for control samples, while Peleg model displayed the most suitable samples irradiated at 1 and 1.5 kGy, then the Enderby model for those treated with 2 kGy. Results of Net isosteric heat of adsorption suggested that high irradiation dose increases the spontaneity of moisture adsorption. Hence, gamma irradiation exhibited a significant effect on the water-specific surface area of which the magnitude was proportional to the increasingly applied doses. This effect was also visibly significant on the optimum water activity [a_w_ (op)] for proper dried fig storage. Indeed, a_w_ was about 0.4243 for control samples, which is much higher compared to irradiated ones (a_w_ = 0.2). Information from this research suggests that gamma irradiation at a dose up to 2 kGy extended the dried figs' shelf life. Since many aspects related to the impact of gamma irradiation on the moisture adsorption isotherms and thermodynamic properties of dried figs as well as in other foods have yet to be further investigated, this study provides interesting results that may be a useful reference for future research direction.

## Introduction

Water plays a key role in food quality and stability during storage due to its ability to interact with other molecules and to affect their conformation, mobility, and functionality (1). Knowledge of the hygroscopic properties of foods is of great importance for their processing, in order to understand and predict the influence of variations in ambient relative humidity on their water content during storage (1). Moisture adsorption isotherms are the first step in scientific understanding of the state of water in food and their derivative compounds, and its potential influence on the many spoilage reactions occurring during their preservation (2). It is an extremely useful tool as a quantitative approach to predicting the final water content to be reached at the end of the drying process. It also helps in the selection of the appropriate packaging materials, ingredients, and optimum storage conditions to ensure the physical, chemical, and microbial stability of the dried products during storage (3). Due to the very complex composition of food products, theoretical predictions are usually inaccurate. Therefore, adsorption isotherms must be established experimentally for each product (4). In this sense, several mathematical models based on more or less physical empirical and/or theoretical criteria can help to describe the relationship between equilibrium water content and equilibrium relative humidity along with temperature (5, 6). These are useful tools that allow a better valuation of the thermodynamic properties for optimal conservation (7).

Figs and particularly dried figs are a traditional pantry staple for healthy eating in Middle Eastern and North African countries as fig trees grow abundantly in such hot and dry climates (8, 9). Despite the importance of this food source, several problems are impacting the marketability of dried figs in markets, mainly insect infestation, microbial growth, and mycotoxin contamination alongside color degradation during storage. Most of the post-harvest chemical fumigants used for dried figs, such as ethylene dibromide, methyl bromide, and ethylene oxide, are either banned or phased out because of their serious adverse impacts on human health and the environment (10–12). As well, based on the health hazards and environmental effects of conventional chemical post-harvest treatments, such as CO_2_ applied under atmospheric conditions and at high pressure (13), magnesium phosphide (Mg_3_P_2_) (14), sulfuryl fluoride (SO_2_F_2_) (15), sulfur dioxide (SO_2_) (16), and phosphine (17), alternative processing techniques are always needed for preserving both dried figs nutritional quality and consumer health.

Studies on gamma irradiation processing at a low irradiation dose (1.0 kGy or less) have been reported to be a suitable quarantine technology for post-harvest pest control (18–20), especially for the disinfestation of dried figs (16). Kabak et al. (21) also reported that irradiation processing can be an effective alternative technology in post-harvest inhibition of microbial contamination and mycotoxin biosynthesis during storage, thus to extend the shelf life of food products. The application of gamma irradiation in the food industry is generally framed on each side by minimum value allowing the desired objective to be achieved, and a maximum value depending on the cost of treatment, and on the other hand, the product's tolerance to radiation (22). Several relevant studies suggest a dose of 1 kGy for killing microorganisms in dried fruits (16, 23), and other studies suggest a dose higher than 1 kGy to enhance its biochemical attributes and shelf life (24, 25). Additionally, as reported by Farkas (26), to achieve desired levels of microbial and parasite control on a commercial scale, it is desirable to use the lowest possible doses necessary, since maintaining organoleptic and nutritional qualities and keeping costs down are also important factors. The focus of the aforementioned studies was on the impact of ionizing radiation on biochemical properties, microbial growth, and post-harvest pest control. However, very few studies have looked at the effect of gamma irradiation processing on the adsorption isotherms of agri-food products. Among these very few studies, only the influence of a single dose (1 kGy or less) of irradiation was investigated (7, 27–29).

Depending on what has been mentioned above, especially according to Cetinkaya et al. (16), Stefanova et al. (22), Ahmed (23), Azelmat et al. (24), and Hussain et al. (25), and as no single previous study has been undertaken on gamma-irradiated dried figs moisture isotherms thus far, we thought to explore this aspect within the range of 1 and 2 kGy for quality maintenance and quarantine purposes. Thus, this research was conducted as the first study investigating the adsorption isotherms and the thermodynamic properties of dried figs, as treated using gamma irradiation processing at increasing doses (0, 1, 1.5, and 2 kGy). The main objective of this work is to report for the first time the thermophysical behavior of moisture adsorption isotherms of irradiated dried figs compared to control, especially the relationship between equilibrium moisture content and corresponding relative humidity at three constant temperatures of 30, 40, and 50°C. Also, to fit the experimental data using Peleg, Enderby, and GAB's mathematical models to determine the one providing the best throughput resolution of equilibrium points with regard to gamma irradiation. The comparison for both controlled and irradiated dried figs of the optimal water activity for storage [a_w_ (op)] and the major thermodynamic properties such as water-specific surface area (S_0_), net isosteric heat (q_st_), Gibbs free energy (ΔG_β_), and differential entropy of adsorption (Delta S), which represent outstanding properties regarding the energy required for adsorption was reported. In addition, the enthalpy–entropy compensation theory for both controlled and irradiated samples was verified.

## Materials and methods

### Raw materials preparation

Fully ripened figs belonging to *Ficus carica* L. “El Qoti Lebied” cultivar, one of the most locally cultivated clones, were collected during the first week of September 2020 from an orchard in the rural township in Chefchaouen, northern Morocco. Selected fruits had uniform size and maturity, with no diseases and visual blemishes. Initial moisture content was determined using the oven-dried method as described by AOAC (30) and was noted to be 78 ± 1% wet basis (w.b.). Afterward, all figs were blanched by immersion in boiling water containing 1 kg Na Cl (99.5°C at normal atmospheric pressure) in 20 L for 15 s, 30 times; Blanching water to fruit ratio was 2 kg: 1 L. Subsequently, fruits were dried in an industrial hybrid dryer (50°C: 20–24% relative humidity; 4 h). The drying was stopped at a moisture content of 25% according to the dried fig commercial quality standards developed by the United Nations Economic Commission for Europe (31). All dried figs, with the same level of water activity of 0.42, were packaged and sealed in polyethylene terephthalate (PET) bags (size: 10 × 6 cm L/W; permeability: 50–100 and 245.83–408.64 cm^3^.μm/m^2^.h. atm for O_2_ and CO_2_, respectively; permeability to water vapor: 16.25–21.25 g.μm/m^2^.h) to serve as replicates; each bag was 250 g. All samples were stored at room temperature, ~22°C before being treated by gamma irradiation and analyzed for their adsorption isotherms.

### Gamma irradiation

Dried figs irradiation was carried out at the Ionization Center of Boukhalef relevant to the National Institute for Agricultural Research (INRA) of Tangier, Morocco. PET bags prepared as described above were divided into four groups each one placed inside a carton box (22.7 cm length by 19 cm width and 5 cm thickness), one of which was chosen as a control. The three boxes left were treated by gamma rays at increasing doses: 1.0, 1.5, and 2.0 kGy of Cobalt radioisotopic source (^60^Co) with a maximum dose rate of 10.87 Gy/min and a minimum dose rate of 9.64 Gy/min. Each treatment was performed in triplicates. Immediately, after the end of the irradiation, control and irradiated samples were stored at room temperature, ~22°C before the investigation of water adsorption isotherms and thermodynamic properties.

**Figure 1:**
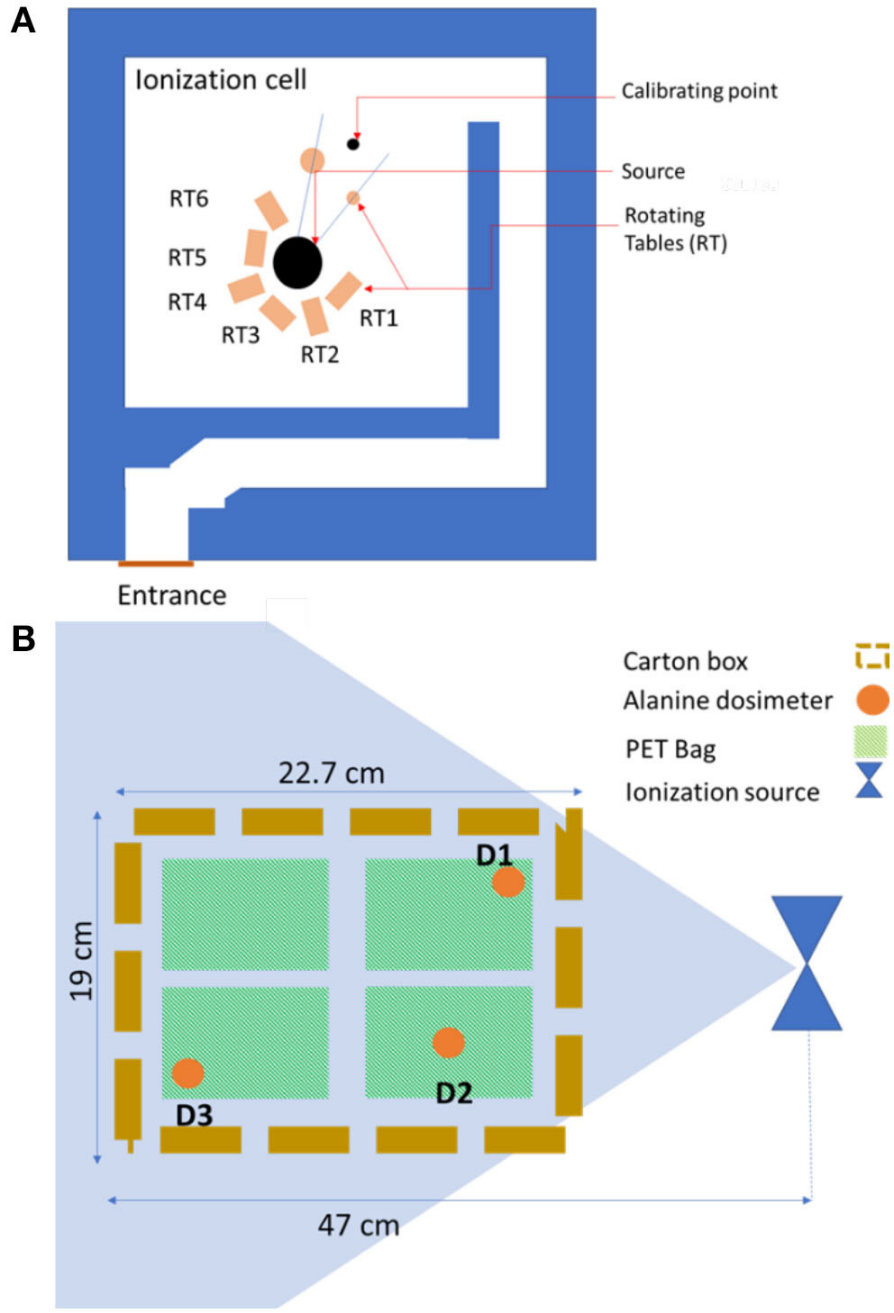
Ionization cell equipped with COB9-60 Cobalt source **(A)** and positioning of the alanine dosimeters in the box of dried figs **(B)**.

Prior to gamma irradiation, the dosimetry was carried out according to a specific protocol, in which the dried figs were presented as described above with four bags (250 g) in each box (1 kg). The source of irradiation is Cobalt 60 COB9 type. The active material is confined inside two successive stainless steel tubular envelopes. These cylinders with a diameter of 10 mm and an active length of 450 mm are closed by massive plugs welded at their ends. The source holder used is made of stainless steel and can hold 22 COB9-type source pens. The unit sources are distributed over a diameter of 140 mm. This source holder allows reloading and use of the sources for several years. The storage container, which is used for the transport of the sources, is type B (U) with a diameter of 870 mm and a height of 1,160 mm. It is made of lead and provides the required protection during transport of the source and during the intervention inside the ionization room. The sources are stored in dry conditions. The ionization cell with a parallelepiped shape 6.1 m long, 5.8 m wide, and 2.6 m high is delimited by walls that ensure the biological protection of operators and the environment (Figure 1A). The concrete walls with a maximum thickness of 1.63 m make it possible to attenuate the radiation up to the limit required by the radiation protection regulations. The location of the three alanine dosimeters placed separately in randomly chosen carton boxes is shown in Figure 1B, which were placed within a 47-cm radius from the ionization source. To ensure that the irradiation of the dried figs was indeed homogeneous and uniform, so as not to have parts irradiated more than others, it was necessary to know the dose uniformity which is in our case 1.129.

### Determination of equilibrium moisture content

The procedures for obtaining absorption isotherms for agri-food products are described by several authors (32–34). In this study, the gravimetric static method at 30, 40, and 50°C with six saturated salt solutions (KOH, MgCl_2_, K_2_CO_3_, NaNO_3_, KCl, and BaCl_2_) was used to determine the equilibrium moisture (X_eq_) values of samples as described by Hssaini et al. (38) and Ouaabou et al. (35) (Table 1). One of the advantages of this procedure is that it allows these salt solutions to generate a well-defined surrounding relative humidity inside the experimental jars and continuously obtain all the relative humidity values in the range from 5 to 90% (36, 37). Each sample was weighted every 2 days until they reached a constant weight value recorded on two consecutive weightings, which were supposed to be the equilibrium. Ten days were required to reach the equilibrium state. The difference of mass before (M_i_) and after equilibrium state (M_f_) at 105°C (±0.1°C) during 24 h gives the equilibrium moisture content (EMC) at hygroscopic equilibrium (X_eq_). It was determined using Equation (1) and reported on a dry weight basis (g water/g dry solid):


(1) $$\begin{array}{l} \text{EMC}={\text{X}}_{\text{eq}}=\frac{{\text{M}}_{\text{i}}\;{-\text{ M}}_{\text{f}}}{{\text{M}}_{\text{f}}} \end{array}$$

**Table 1:** Standard values of the water activities of the six saturated salts as a function of temperature used for the determination of the adsorption curves (35–37).

**Temperatures**	**Values of the water activities**					
30°C	0.0738	0.3238	0.4317	0.7275	0.8362	0.898
40°C	0.0626	0.3159	0.423	0.71	0.8232	0.891
50°C	0.0572	0.3054	0.4091	0.6904	0.812	0.8823
	KOH	MgCl_2_.6H_2_O	K_2_CO_3_	NaNO_3_	KCl	BaCl_2_.2H_2_O

### Modeling of the adsorption isotherms

Although there are several mathematical models to describe the characteristics of moisture adsorption isotherm, Peleg (39), Enderby (40), and GAB's (41) mathematical models (Table 2) are among the most used in the literature and were retained to adjust the experimental data in this study to define the most suitable model, which describes the relationship between the equilibrium moisture content, the water activity, and the temperature. Generally, the problem of modeling the adsorption isotherms consists in finding a function checking the following Equations (2) and (3):


(2) $$\begin{array}{lll} {\text{X}}_{\text{eq}} & = & \text{f}\left[\frac{{\text{P}}_{\text{vp}}}{{\text{P}}_{\text{vs}}}\right]=\text{f}\left({\text{a}}_{\text{w}},\text{T}\right) \end{array}$$

(3) $$\begin{array}{lll} {\text{a}}_{\text{w}} & = & \frac{{\text{P}}_{\text{vp}}}{{\text{P}}_{\text{vs}}}=\frac{\text{RH }\left(\text{\%}\right)}{100} \end{array}$$

where P_vp_: Partial pressure of water vapor in air (Pa), P_vs_: Partial pressure of saturated vapor (Pa), and RH: Relative air humidity (%).

**Table 2:** Moisture sorption isotherm models selected to analyze data for dried figs.

**Models**	**Mathematical expressions**	**References**
Peleg	$X_{eq}=A{\left(a_{w}\right)}^{C\;}+B{\left(a_{w}\right)}^{D}$	(39)
Enderby	$X_{eq}=\left[\frac{A}{\left(1-Ba_{w}\right)}+\frac{C}{\left(1-Da_{w}\right)}\right]a_{w}$	(40)
Guggenheim–Anderson–De Boer (GAB)	$X_{eq}=\frac{ABC\;a_{w}}{\left[1-Ba_{w}\right]\left[1-Ba_{w}-BCa_{w}\right]}$	(41)

A, B, C and D are the models constants.

X_*eq*_ : Equilibrium moisture content [dry basis (kg/kg d.b.)].

a_*w*_ : Water activity.

If the product is in hygroscopic equilibrium with the surrounding air, the water activity (a_w_) is identical to the equilibrium relative humidity (a_w_ = ERH).

The aforementioned models are compared based on the correlation coefficient (*R*^2^) and the standard error of estimated moisture (SE) (42). Curve Expert 3.1 and Originlab pro 8.1 software were used to determine the parameters of selected models using non-linear regression. *R*^2^ (Equation 4) (27) and SE (Equation 5) (38) were used to determine the prediction accuracy of each model to the experimental equilibrium moisture content and water activity data. The best-fitted equation was considered to be the one giving the smallest SE coupled to the highest *R*^2^ value (2, 38, 43–45):


(4) $$\begin{array}{lll} \text{R} & = & \sqrt{\frac{\sum_{\text{i}=1}^{\text{N}}{\left(\text{M}{\text{R}}_{\text{pre},\text{i}}-{\bar{\text{MR}}}_{\text{exp},\text{i}}\right)}^{2}}{\sum_{\text{i}=1}^{\text{N}}{\left(\text{M}{\text{R}}_{\text{exp},\text{i}}-{\bar{\text{MR}}}_{\text{exp},\text{i}}\right)}^{2}}}\; \end{array}$$

(5) $$\begin{array}{lll} \text{SE} & = & \frac{\sum_{\text{i}=1}^{\text{N}}{\left(\text{M}{\text{R}}_{\text{pre},\text{i}}-\text{M}{\text{R}}_{\text{exp},\text{i}}\right)}^{2}}{\text{df}} \end{array}$$

where MR_exp_: The i^th^ experimental equilibrium moisture content, Mi_pre_: The i^th^ predicted equilibrium moisture content, N: The number of observations, and df: The number of degrees of freedom of regression model.

### Adsorption-specific surface area (S_0_)

By using the values of the monolayer moisture contents obtained by GAB model, the values of the water-specific surface area of adsorption can be calculated using Equation (6), and this is expressed in m^2^.g^−1^ of solids (4):


(6) $$\begin{array}{l} {\text{S}}_{0}={\text{M}}_{\text{m}}\times \frac{1}{\text{P}{\text{M}}_{\text{H}2\text{O}}}\times {\text{N}}_{\text{o}}\times {\text{A}}_{\text{H}2\text{O}}=3.5\times 10^{3}\times {\text{M}}_{\text{m}} \end{array}$$

where S_o_: Water-specific solid surface area of adsorption (m^2^.g^−1^ solids); M_m_: Monolayer moisture content (g/100 g, d.b); N_o_: Number of Avogadro (6 × 10^23^ molecules per mole); A_H2O_: Area of a water molecule (10.6 x 10^−20^ m^2^); and PM_H2O_: Molecular weight of the water (18 g.mol^−1^).

### Optimal water activity [a_w_ (op)] for storage

Food preservation by dehydration is based on the principle to maintain the water activity (a_w_) below the critical threshold where microbial growth along with some undesirable chemical reactions occur (46). It is well-known that each microorganism has a critical a_w_ below which growth cannot occur. For instance, pathogenic microorganisms cannot grow at a_w_ < 0.86; yeasts and molds are more tolerant but usually, no growth occurs at a_w_ < 0.62. The so-called intermediate moisture foods (IMF) have aw values in the range of 0.65–0.90. Generally, with a_w_ between 0.4 and 0.2, the product is most stable with respect to lipid oxidation, non-enzymatic browning, and enzyme activity (41, 47). Among the objectives of the moisture isotherms study of a dehydrated agri-food product, is that it provides precise information on the equilibrium moisture content to be reached at the end of drying, and how to handle the product during packaging and storage for proper preservation. For this purpose, the optimal water activity values for conservation [a_w_ (op)] of irradiated and control dried figs were determined. Indeed, the adsorption isotherms curves can be described as a polynomial equation of the third degree (48). The zone of better stability of the products corresponds to the central part or “plate.” This calculation method consists of making a polynomial decomposition of equilibrium moisture content, for all the experimental results of each sample, according to the water activity. Therefore, the optimum relative humidity for storage can be determined by calculating the value for which the second derivative of X_eq_ is canceled or “inflection point” (7, 49).

### Net isosteric heat of adsorption (q_st_) and differential entropy of adsorption (ΔS)

The calculation of the isosteric heat of adsorption is defined by the sum of the net isosteric heat of adsorption (q_st_) and the latent heat of condensation of pure water vapor at system temperature (H_v_) (50) and is given by Equation (7):


(7) $$\begin{array}{l} {\text{Q}}_{\text{s}}={\text{q}}_{\text{s}}+{\text{H}}_{\text{v}} \end{array}$$

From the experimental data of the adsorption isotherms, the net isosteric heat of adsorption at constant humidity (X_eq_) can be calculated using the Clausius–Clapeyron equation (Equation 8) (51):


(8) $${\text{q}}_{\text{st}}=-\text{R }{\left[\frac{\text{d }({\text{ln}}_{\left({\text{a}}_{\text{w}}\right)})}{\text{d}(\frac{\text{1}}{\theta })}\right]}_{{\text{X}}_{\text{eq}}}$$

where a_w_: Water activity, θ: Absolute temperature (K), and R: Ideal gas constant (8,314 × 10^−3^ kJ.mol^−1^.K^−1^).

The value of water activity as a function of a fixed equilibrium moisture content can thus be determined from this equation using adsorption isotherms at different temperatures. For this, we assume that the net isosteric heat of adsorption is independent of temperature, and so Equation (8) can be integrated to give Equation (9):


(9) $$\ln({\text{a}}_{\text{w}})=\frac{{-\text{q}}_{\text{st}}}{\text{R}}\frac{\text{1}}{\text{T}}\text{+}\frac{\Delta \text{S}}{\text{R}}$$

where ΔS : Differential entropy of sorption (J.mol^−1^.K^−1^).

### Gibbs energy (ΔG_β_)

From the slope of the curve ln_(_*a*__*w*_)_= f ($\frac{1}{T}$), the net isosteric heat of sorption (q_st_) was determined. The deduced slope $\frac{q_{st}}{R}$ and the constant of $\frac{\Delta S}{R}$ displayed a linear relationship. Indeed, referring to Equation (10) below, Gibbs specific energy can be calculated:


(10) $$\Delta {\text{G}}_{\beta }{=-\text{RTLn}}_{\left({\text{a}}_{\text{w}}\right)}$$

Between q_st_ and ΔS, the theory of enthalpy–entropy compensation proposes a linear relationship given by Equation (11) (52):


(11) $$\begin{array}{l} {{\text{q}}_{\text{st}}=\text{T}}_{\beta }\text{S}+{\Delta \text{G}}_{\beta } \end{array}$$

where T_β_: Isokinetic temperature is the temperature at which all sorption reactions proceed at the same rate, ΔG_β_ : Gibbs energy which helps to determine whether the adsorption process is spontaneous or not. The system is spontaneous if ΔG_β_ is negative (–ΔG_β_) and conversely if ΔG_β_ is positive (+ΔG_β_).

The validation of the compensation theory is tested by comparing the isokinetic temperature to the harmonic mean temperature (T_hm_). The T_hm_ was determined as follows:


(12) $$\begin{array}{l} T_{hm}=\frac{n}{\sum_{1}^{n}\left(\frac{1}{T}\right)} \end{array}$$

The enthalpy–entropy compensation theory can be applied only if T_β_ ≠ T_hm_.

## Results and discussion

### Moisture adsorption isotherms

**Figure 2:**
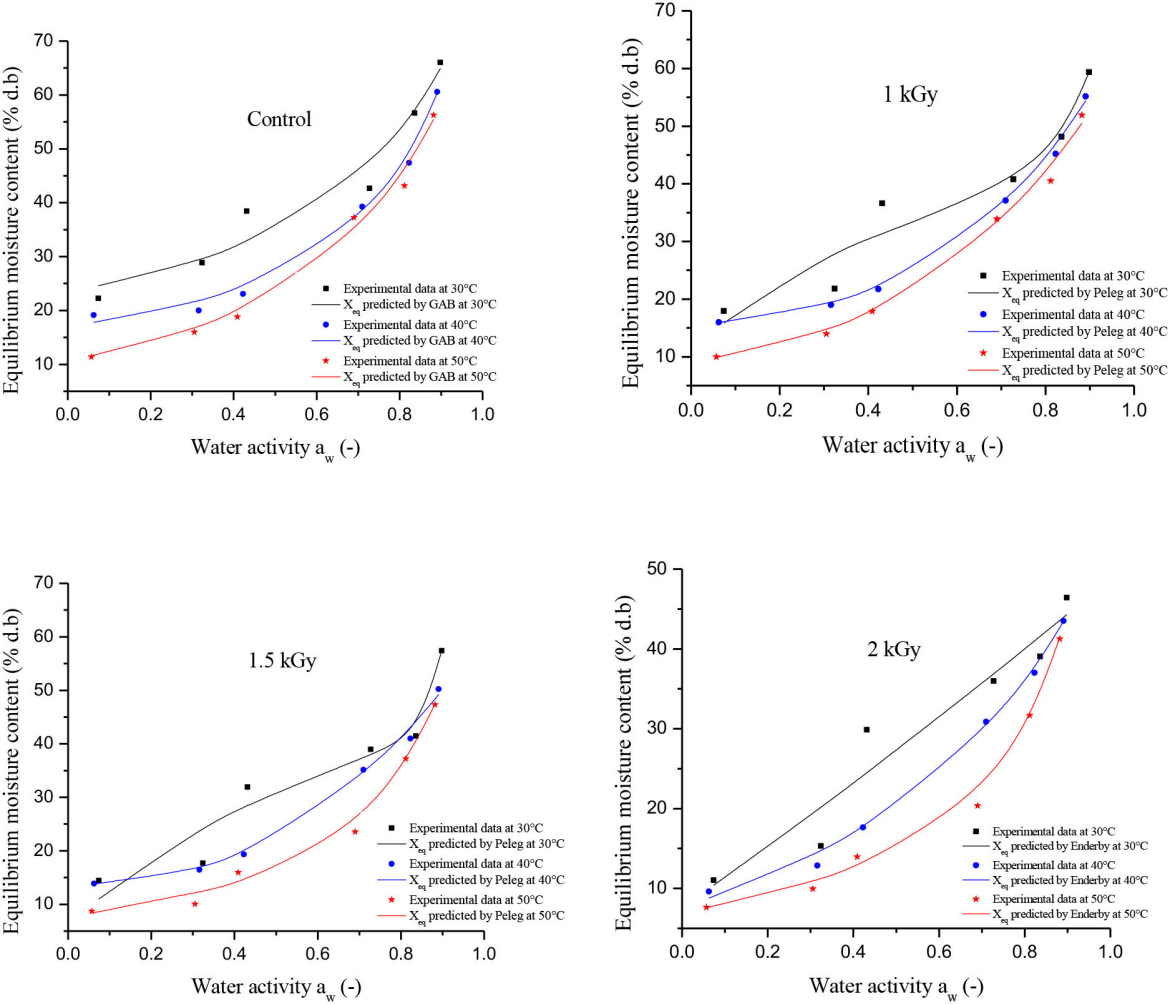
Adsorption isotherms of control and gamma irradiated (1, 1.5, and 2 kGy) at different temperatures.

Figure 2 illustrates the effect of temperature on the equilibrium moisture content (EMC) of control and irradiated dried figs. At each water activity, the EMC plotted in the Figure 2 represents the average of triplicate measurements. Since it was difficult to read the moisture equilibrium content kinetic as a function of aw (because of some overlapping points), we thought that it would be important to support that with numerical data. Thus, Table 3 reports the experimental data for the equilibrium moisture contents of control and gamma-irradiated dried figs. With the reference to the data reported in Table 3, we notice that at constant water activity, moisture adsorption at the different temperatures applied displayed significant decreases in equilibrium moisture levels X_eq_ (% d.b) for all dried fig samples as the temperature rises. This trend becomes more important as water activity decreases. For instance, at a constant a_w_, the equilibrium moisture content for 1 kGy irradiated sample on a K_2_CO_3_ supersaturated saline solution were, respectively, 36.60, 21.71, and 17.89 g water/g d.b at 30, 40, and 50°C. Likewise, EMC levels for 1.5 kGy irradiated samples on BaCl_2_.2H_2_O solution were, respectively, 57.41, 50.20, and 47.28 for the following a_w_ values 0.898, 0.891, and 0.882 (Table 4). However, it is noteworthy that, at 30°C it was observed a sharp increase in EMC within the range of a_w_ between 0.32 and 0.43, which is equivalent to 3 times the EMC increase that occurred under 40 and 50°C. This irregular increase trend was for both control and irradiated samples but was not observed under other temperatures. This could be linked probably to the higher active state of water molecules at high temperature levels, thus the attractive forces between them decreased. Thus, the EMC increased with decreasing temperature at constant relative humidity. The same pattern was observed in the study by Hssaini et al. (38) over fig samples (c.v “Nabout” and “Sarilop”). Regarding the overall EMC trend following variant a_w_ and temperature, Hidar et al. (49) reported that it means that the material becomes less hygroscopic as the attraction strength between water molecules is reduced. Farahnaky et al. (2) reported with reference to Chowdhury et al. (44) and Jamali et al. (45) that as the temperature increases, water molecules become less stable and dissociate from the water binding sites of the food material, thus reducing the moisture content of the monolayer. Other similar investigations have reported that this is most often typical of agri-food materials (27, 28).

**Table 3:** Equilibrium moisture content *X*_eq_ (% d.b) data of control and gamma irradiated (1, 1.5, and 2 kGy) dried figs in adsorption at 30, 40, and 50°C.

**a_w_**	**Control**	**1 kGy**	**1.5 kGy**	**2 kGy**
**Sorption isotherms at 30** **°C**				
0.0738	22.2300	17.9473	14.4153	11.0383
0.3238	28.8623	21.8213	17.6886	15.3081
0.4317	38.4398	36.6062	31.9034	29.8778
0.7275	42.6365	40.7842	38.9702	35.9780
0.8362	56.6191	48.1815	41.4434	39.0498
0.898	66.0061	59.3658	57.4161	46.4088
**Sorption isotherms at 40** **°** **C**				
0.0626	19.1460	15.9733	13.8532	9.5933
0.3159	20.0140	18.9915	16.4681	12.8722
0.423	23.0670	21.7163	19.3168	17.6189
0.71	39.2474	37.0987	35.1512	30.8564
0.8232	47.4143	45.1684	40.9511	37.0010
0.891	60.5485	55.1379	50.2033	43.5096
**Sorption isotherms at 50** **°** **C**				
0.0572	11.3813	9.9799	8.6962	7.6092
0.3054	15.9682	13.9664	10.0488	9.9535
0.4091	18.8157	17.8946	15.9337	13.9468
0.6904	37.2508	33.8758	23.5558	20.3438
0.812	43.1138	40.4748	37.1860	31.6761
0.8823	56.2481	51.8870	47.2790	41.2598

**Table 4:** Statistics (R^2^ and SE) of sorption mathematical models (Peleg, Enderby and GAB) for control and gamma irradiated (1, 1.5, and 2 kGy) dried figs at 30, 40, and 50°C.

	**Temperature**		**Models' constants**				**R^2^**	**SE**
			**A**	**C**	**B**	**D**		
Control	30	Peleg	57.95	8.0684	43.445	0.2659	0.9889	3.8785
	40		19.9258	0.01635	58.684	3.4451	0.993	2.2523
	50		54.4522	2.7454	16.192	0.1257	0.994	3.0817
	Average						0.99	3.07
	30	Enderby	6.1394	0.9392	862.6139	−26.5349	0.9872	4.1684
	40		−701.7006	0.1223	757.0997	0.1223	0.8888	12.3081
	50		−41.0987	0.3456	82.7602	0.3456	0.9642	7.4538
	Average						0.95	7.98
	30	GAB	0.07752	23.753	1.2795		0.9773	4.4762
	40		0.07602	14.6948	1.3403		0.9956	1.9706
	50		0.07594	207.4609	0.07877		0.9941	2.5029
	Average						0.99	2.98
1 kGy	30	Peleg	62.8452	12.3153	44.4566	0.3948	0.9708	5.9507
	40		19.2505	0.0684	50.5187	3.1025	0.9987	1.3014
	50		50.4786	2.7289	14.9247	0.1445	0.996	2.3435
	Average						0.99	3.2
	30	Enderby	510.321	−17.0761	5.87	0.9229	0.9678	6.2404
	40		750.4792	0.0744	−696.6964	0.0745	0.9206	9.8256
	50		16.4457	0.7485	11.3794	−13.4896	0.9965	2.1987
	Average						0.96	6.09
	30	GAB	0.0673	44.6348	0.6354		0.9605	5.638
	40		0.0659	20.9003	0.8509		0.9983	1.2072
	50		0.0662	11.9418	0.0693		0.9958	1.9601
	Average						0.98	2.94
1.5 kGy	30	Peleg	45.1958	0.5429	3034.3954	49.4879	0.9724	5.9059
	40		47.0705	2.4667	13.7966	0.000346	0.9972	1.7597
	50		15.5316	0.2158	58.7568	4.7689	0.9953	2.3641
	Average						0.99	3.34
	30	Enderby	2.9384	1.0098	258.1852	−7.9319	0.963	6.8213
	40		12.2867	−1.0405	13.7914	0.7609	0.9957	2.1719
	50		9.5263	0.8943	2.0563	−2.6424	0.9956	2.3038
	Average						0.98	3.77
	30	GAB	0.057249	29.7847	0.7092		0.9531	9.258
	40		0.052985	23.2266	0.3994		0.996	1.7271
	50		0.05061	12.8885	0.6863		0.9956	1.8753
	Average						0.98	4.29
2 kGy	30	Peleg	0.0003	−4.6034	48.1378	0.8078	0.9684	5.4669
	40		−0.7838	1.038	12.6242	1.038	0.9678	5.5225
	50		0.0152	0.2528	52.8631	5.4738	0.9975	1.4747
	Average						0.98	4.15
	30	Enderby	2.7997	−3.6677	37.4636	0.0925	0.9659	5.6752
	40		2.4187	−3.1361	16.6089	0.658	0.9978	1.4362
	50		7.142	0.9204	11.882	−0.148	0.9974	1.523
	Average						0.99	2.88
	30	GAB	0.04099	0.5241	0.0636		0.9491	5.6357
	40		0.03968	0.5189	0.0572		0.9961	1.5743
	50		0.03212	0.511	0.0089		0.9972	1.2712
	Average						0.98	2.83

**Figure 3:**
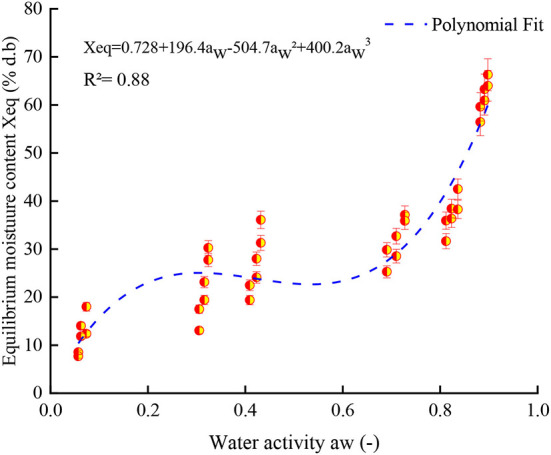
Equilibrium moisture content of 1 kGy treated samples under all investigated temperatures with the perfect s-shaped curve of adsorption isotherm.

**Figure 4:**
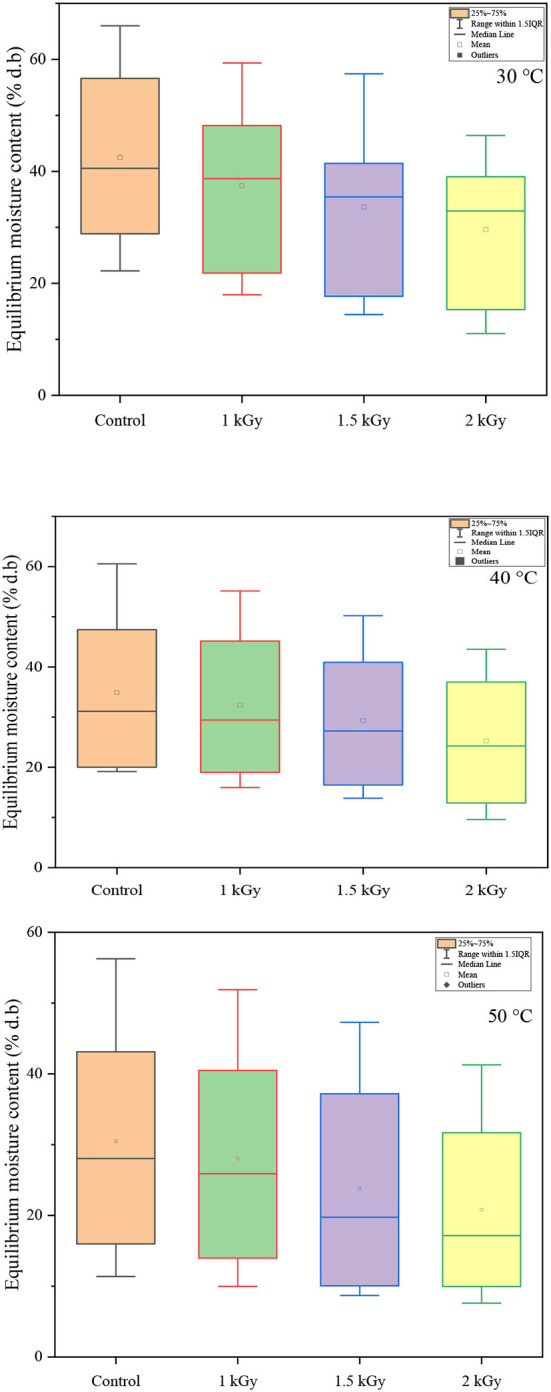
Boxplots showing the decreasing pattern of equilibrium moisture content (% d.b) with the increase of irradiation dose under each investigated temperature.

According to Brunauer's classification (53) and the International Union of Pure and Applied Chemistry (IUPAC) classification, data in this study obtained for both controlled and irradiated dried figs adsorption isotherms are classified as type II, which is most likely close to the sigmoidal shape. In this sense, Figure 3 illustrates the equilibrium moisture content of 1 kGy treated samples under all investigated temperatures and perfectly shows the s-shaped curve. More interesting, data in this study reported displayed a remarkable influence of gamma irradiation on the equilibrium moisture contents and the adsorption isotherms of samples within the entire range of water activity and temperature. In this sense, a boxplot graph of data under each tested temperature has been plotted and illustrated in Figure 4. Thus, gamma irradiation impacted very significantly the equilibrium moisture contents values and the adsorption isotherms, of which the equilibrium moisture content decreased proportionally as the irradiation dose increased. Indeed, irradiated samples had a smaller water adsorption capacity compared to control dried figs due to the possible influence of the gamma rays. This behavior can be explained by the fact that gamma rays probably interact with atoms and/or molecules in the cell considering the penetrating depth and radiolysis capabilities (27, 54, 55). In addition, it is possible that this interaction is a threshold event as a function of the gamma irradiation dose. As irradiation dose increases, water molecules and other structural macromolecules are affected. The less stable products break away from the water-binding sites of the food material, reducing the moisture content of the monolayer or even multilayer and interstitial (27).

However, because this is the first time that this very particular aspect has been highlighted and that unfortunately there is no report to our knowledge on irradiated dried figs isotherms or other similar biological commodities, the behavior and response to gamma irradiation of biological matrices different from dried figs were analyzed due the fact that the behavior and the response of these matrices could be likely to present similar behaviors vis-à-vis gamma irradiation. Thus, our results are slightly different from the study by Mghazli et al. (27), which reported that control and irradiated samples had the same hygroscopic behavior. Also, in that investigation, in water activity ranging between 0.2 and 0.7, the equilibrium moisture content of control samples was higher than the irradiated samples. Moussaoui et al. (7) and Lahnine al. (28) have observed the same findings. However, these results can be explained by the low ^60^Co γ irradiation dose (< 1 kGy) applied, which may have no significant impact on the stability of the product.

### Adsorption isotherms modeling

The results of fitting the experimental data into Peleg, Enderby, and GAB sorption equations are shown in Table 4. The values of the correlation coefficient (*R*^2^) and the standard error (SE) of estimated moisture are compared for each model to provide the best-fit model. From Table 4, tested models have generally expressed an average value of *R*^2^ of 0.98 coupled with an average SE of 3.96 for all samples. According to SE, the values showed that the models used were in agreement with the experimental data with the exception of Enderby, which showed errors >5, with regard to equilibrium points of the control. After analyzing the statistical parameters in Table 4, the fit using GAB model was the most suitable for describing the adsorption isotherms of the control. On the one hand, the model has combined the highest *R*^2^ (0.99) and the lowest SE (2.98) within the temperature range investigated in this study. On the other hand, the model Peleg displayed the best fit for irradiated dried figs at the first two irradiation doses 1 and 1.5 kGy. Obviously, this model displayed the highest *R*^2^ (0.99), which was similar for both doses coupled with the lowest SE levels (3.2 and 3.34, respectively). The fit using the Enderby model was most appropriate to describe the adsorption isotherms for dried figs irradiated at the 2 kGy doses. The performance of the model selected for control and gamma-irradiated dried figs at different temperatures is illustrated in Figure 2.

These results join other previous studies which reported that from the criteria indices *R*^2^ and SE, the aforementioned models could be useful for the ERH prediction of dried figs with the highest fit resolution (56). However, these results are slightly opposite to those reported by Hssaini et al. (38) who found that Enderby and Peleg showed the best prediction of adsorption isotherms for two varieties of figs “Kadota” and “Sarilop.” In addition, the gamma irradiation used in this study displayed a remarkable effect on the fig adsorption isotherms modeling within the entire range of temperature investigated in this study. Thus, considering the fitting statistics parameters, it appears that at 50°C, *R*^2^ increases and the SE decreases with increasing irradiation dose, particularly in the case of the GAB model experimental data. From the fact that the combination of higher *R*^2^ and lower SE determines the goodness of data modeling, the quality of the fit of the three models was slightly greater than those of the control at 40 and 50°C considering increasing doses of irradiation. Nevertheless, despite the multitude of mathematical models used to describe the adsorption isotherms of several foods, none of them can accurately predict moisture isotherms over the entire range of water activity and for all food matrices (44, 57).

### Water-specific surface area of adsorption (S_0_)

Adsorption is the phenomenon of the attraction and retention of molecules from a liquid or gas “adsorbate” by a solid “adsorbent” at its surface, which leads to a higher concentration of molecules at the surface. Several factors will therefore influence this phenomenon, among others: particle size, mass of adsorbent, pH, and temperature of the solution. The particle size of the adsorbent is a parameter closely related to the specific adsorption surface area; small particles have a large specific adsorption surface area and therefore work much better than large particles. In addition, if the adsorption is favored by the low temperatures of the solution, it is exothermic, otherwise, it is endothermic (58). Table 5 reports the values of the water-specific surface area of adsorption calculated using Equation (6), and the monolayer moisture contents obtained by GAB model. According to the literature, the value of the water sorption–specific surface area of a food product is ranged between 100 and 250 m^2^.g^−1^ and is associated with the amount of polymers present in the food (4, 59). Considering those mentioned above, and after analyzing the statistical data in Table 5, we can conclude that the temperature significantly affects the interaction between the water and solid surface area. Indeed, most likely, the increase in temperature is able to change the structure of proteins and improve the interaction of proteins with other components, such as carbohydrates and fats; therefore, the available water adsorption sites have decreased due to the hydrophobicity of the proteins (60). Furthermore, based on the results, gamma irradiation has impacted very significantly the water-specific surface area between the control and treated samples. Thus, as the gamma irradiation dose increases, the effect tends to be more pronounced. Indeed, at a constant temperature, the S_0_ has significantly decreased as the gamma irradiation dose increased, until it reaches approximately half the water-specific surface area of control samples. This trend becomes more important as the temperature increases. In fact, both temperature and gamma rays effectively contribute to dimmish the S_0_ of dried figs in comparison to untreated samples.

**Table 5:** Water specific surface area (S_0_) of control and gamma irradiated (1, 1.5, and 2 kGy) dried figs at 30, 40, and 50°C.

**T (°C)**	**Control**	**1 kGy**	**1.5 kGy**	**2 kGy**
30	271.32	235.55	200.3715	143.465
40	266.07	230.65	185.4475	138.88
50	265.79	231.7	177.135	112.42

These findings join the other previous studies reported by Lahnine et al. (28) and Moussaoui et al. (29), who stressed the same patterns as described above. Likewise, according to Calzetta Resio et al. (61), the existence of an intrinsic microporous structure in the matrix of many biopolymers is probably behind the large adsorption surface. As reported in Table 5, as the temperature increases the value of S_0_ decreases. Considering this hypothesis mentioned by Calzetta Resio et al. (61), it can be assumed that gamma irradiation probably decreases intrinsic microporous structures depending on the intensity of the dose. Indeed, at higher temperatures, water molecules easily dissociate from water-binding sites. Moreover, the distance, attraction, and states of molecules also vary as the temperature changes (60). These behaviors could be more pronounced as the intensity of the irradiation dose increases assuming that physicochemical changes can occur strongly in the product, which very strongly reduces the number of active sites for the binding of the water. There is, however, great complexity in the response that a matrix can generate to gamma radiation. This can affect both the specific surface area and the cells to varying degrees depending on the dose of irradiation applied (62).

### [a_w_ (op)] for storage conditions

Table 6 reports adsorption curves experimental data of control and gamma-irradiated dried figs according to a polynomial model of 3rd degree. The optimal water activity found in this study concords very slightly with the studies conducted by Moussaoui et al. (7), Mghazli et al. (27), and Lahnine et al. (28). Thus, the control sample presents the higher optimal water activity values (0.4243) compared to irradiated samples. Indeed, the irradiation dose significantly influenced the optimal values of water activity between the applied doses, particularly between 1 and 1.5 kGy treated figs, where the deduced values were 0.2865 and 0.2636, respectively. These doses were half those found in the control (0.4243). It is noteworthy that a very slight difference was spotted between 1.5 and 2 kGy irradiated, which a_w_ have barely changed (0.2636 and 0.2634, respectively). The findings are slightly different from the investigation conducted by Mghazli et al. (27) who reported opposite results. Thus, the control samples presented less water activity (0.39) than the irradiated samples (0.40). This behavior could be associated with the low gamma irradiation dose (< 1 kGy) applied, which may have not expressed any significant impact on the stability of the product.

**Table 6:** Analysis of the curves of adsorption according to a polynomial model of 3rd degree.

**Samples**	**[*a_*w*_* (op)]**
Control	0.42
1 kGy	0.29
1.5 kGy	0.26
2 kGy	0.26

Among the main components of food matrices, besides water, carbohydrates, proteins, fats, vitamins, and minerals, also play a crucial role in human nutrition with essential major interest (63). For many years, the effects of irradiation on these components have been studied and are still explored today in a wide range of food products because the ionizing effects of radiation on food are highly dependent on the composition of the matrix and cannot be assumed similar to those observed in each individual component irradiated separately (64). This technology induces certain primary effects in food matrices that occur in particular due to the presence of water molecules by ionization and/or excitation, which increases exponentially by the secondary action of free radicals which can result from stress on the matrix (65). These chemically highly reactive species have the ability to interact with each other and/or with other food components, leading to the formation of new molecules which are not present in unirradiated foods (66).

In addition, among the various parameters which modify the radical species yield, probably the dose rate, the nature and energy of the irradiation, more generally the irradiation dose could hold a major role. Thus, these chemically highly reactive species are thus likely to add their effects to those of irradiation on cells and to modify probably the metabolism and the water retention capacity and potentially water activity on the matrix. As described by Farkas (67), food preservation is generally based on the removal of moisture which inhibits the development of pathogenic microorganisms and also minimizes fruit spoilage reactions triggered by moisture, among other enzymatic and Maillard browning reactions. However, the moisture content alone is not sufficient to predict the stability of foods, as it is not the total moisture content, but rather the water activity that determines the shelf life of food (47). Knowing as mentioned above that the a_w_ requirements of the various microorganisms vary considerably, our results in this study reported showed a significant effect of gamma irradiation on the decrease in the activity of water as a function of the dose absorbed, from 0.4 (untreated dried figs) to 0.2 (irradiated dried figs). This may suggest the preservative effect of the gamma irradiation technology in extending the shelf life of dried figs in relation to the water activity, since at a_w_ between 0.4 and 0.2, the product is also the most stable with regard to lipid oxidation, non-enzymatic browning, and enzymatic activity (41).

### Net isosteric heat of adsorption (q_st_)

**Figure 5:**
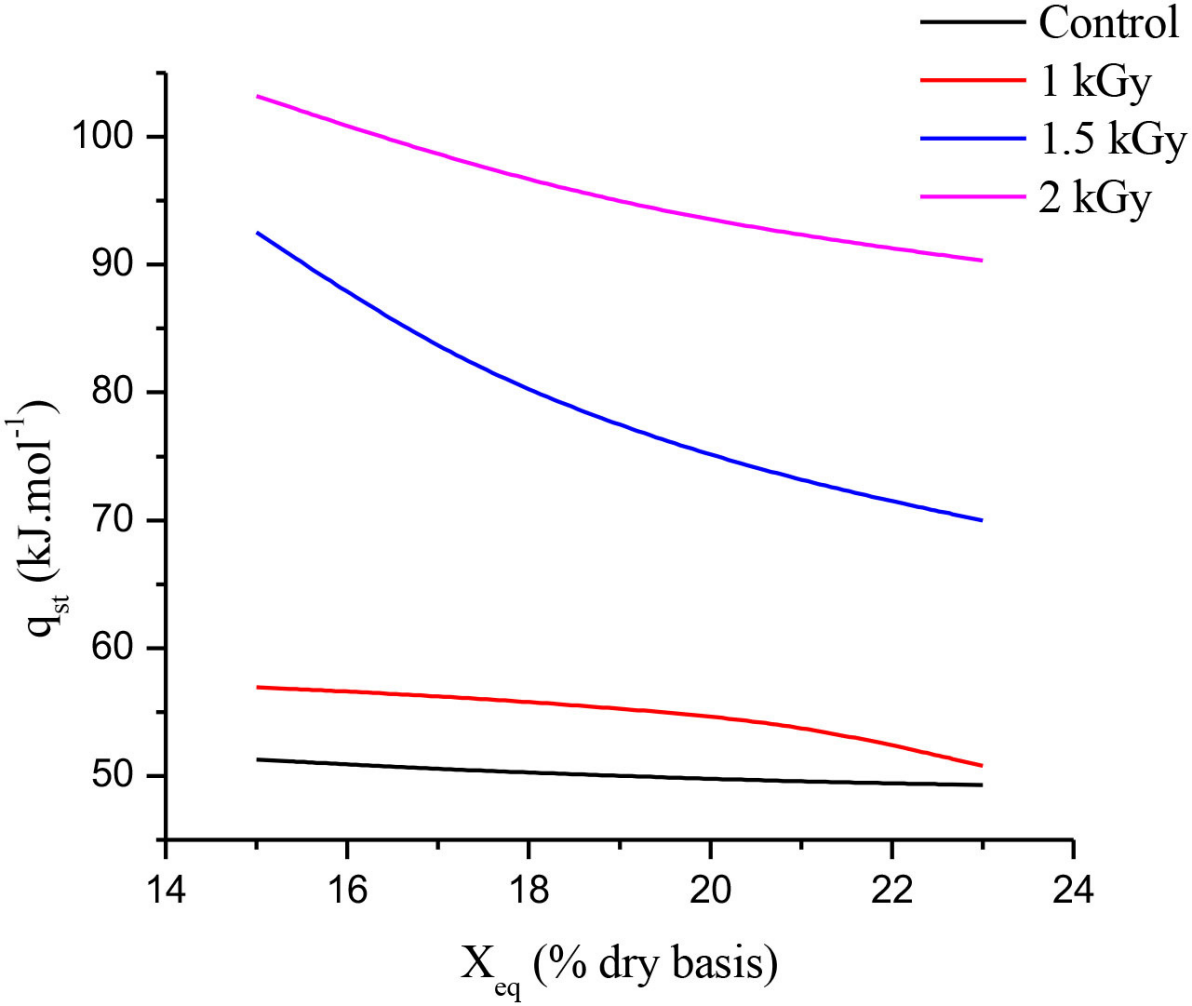
Relationships between equilibrium moisture content (X_eq_) and net isosteric heat of adsorption (q_st_).

To fix water molecules to the food product, net isosteric heat (q_st_) represents the energy required at system temperature (68). Figure 5 illustrates the net isosteric heat of adsorption for control and gamma-irradiated dried figs and shows strong relationships between equilibrium moisture content (X_eq_) and net isosteric heat of adsorption (q_st_). Indeed, with increasing equilibrium moisture content, the strength of water molecules binding with the solid material decreases; therefore, as the moisture content increases, the net isosteric heat of adsorption decreases (69). As shown in Figure 5, at the lowest values of the X_eq_, the q_st_ takes the highest values. Additionally, as the X_eq_ increases, the q_st_ decreases for all samples, illustrating the strong bond of water with the matrix. This result is similar to that reported by Lahnine et al. (28). Once again, the gamma irradiation effect over dried fig net isosteric heat of adsorption displayed significant differences in q_st_ patterns following the increasing irradiation doses. Thus, at a constant temperature, as the gamma irradiation dose increase, the energy required for moisture adsorption increases, and therefore, the samples become more humidified. Nevertheless, the energy required for control samples is lower than for irradiated dried figs. This result is in agreement with the hypothesis of Moraes et al. (70), according to which the energy connecting water molecules to primary sorption sites becomes greater than the energy connecting water molecules when humidity is low. This behavior suggests that irradiated samples will have probably a great storage ability compared to control samples. Indeed, 2 kGy treated samples needed almost twice the energy needed by the control sample to absorb moisture.

Among the molecules of a plant matrix, those of water interact greatly with gamma rays due to the ubiquity of water in the plant cell (65, 71). Ionization, dissociation, and excitation are the main effects of gamma radiation. Low interaction can result from excitation, while ionization and dissociation are likely to result in strong interaction. This results in the production of radical species described as the primary effect of radiation (63). Due to the high reactivity of these chemicals' species, side effects will occur probably to potentially modify the structure and the stability of the matrix at different levels depending on the irradiation dose (64, 71). These radical species can damage or modify important components of the matrix, such as chloroplasts, and also modify biochemistry in carbohydrate transport mechanism (72), starch–sugar interconversion (73), dilation of thylakoid membranes and altered photosynthesis in cell structure (65), and texture change of some foods where polysaccharides such as pectin can be broken down leading to the release of calcium, causing the product softness increase (64, 65). Owing to these effects, alongside others, it is probably possible that the natural resistance of certain cells and irradiated membrane tissues increases, necessitating greater fixing energy for the water to the food matrix.

### Differential entropy of adsorption (ΔS)

**Figure 6:**
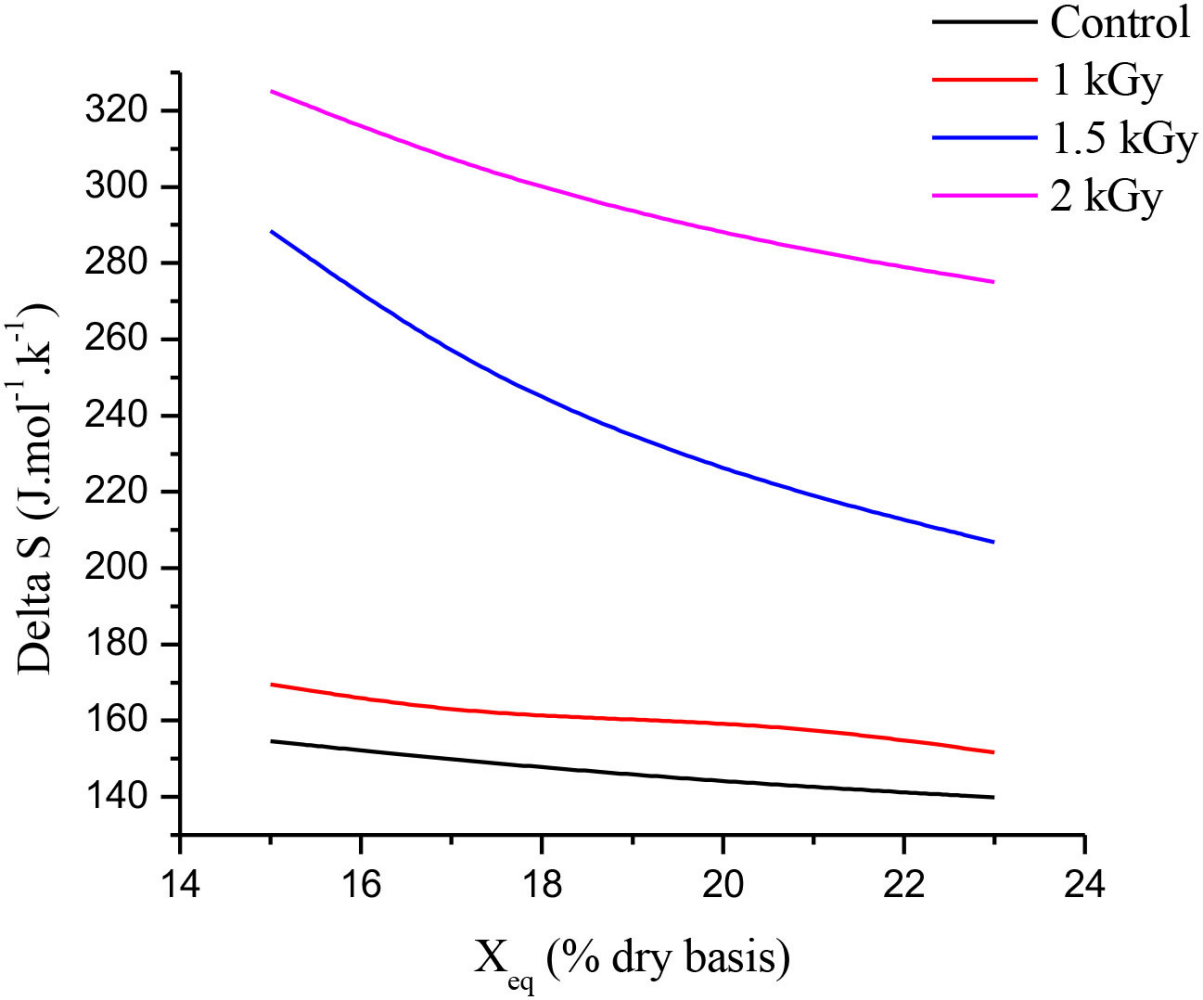
Relationships between equilibrium moisture content (X_eq_) and differential entropy of adsorption (ΔS).

Figure 6 illustrates the relationships between equilibrium moisture content (X_eq_) and differential entropy (ΔS) of control and gamma-irradiated dried figs at doses 1, 1.5, and 2 kGy. According to Madamba et al. (74), at a given energy level, the differential entropy of a material is proportional to the number of sorption sites available. From Figure 6, as the humidity increases, the differential entropy decreases for all samples. This behavior of the differential entropy shows once again, a strong dependence on humidity and on the dose of gamma irradiation applied. Thus, the differential entropy of 2 kGy treated samples is twice that of control samples. Obviously, further analysis is required to deepen our understanding of changes occurring at the molecular scale according to irradiation with regard to dried products' differential entropy of adsorption.

### Gibbs energy (ΔG_β_)

Within a confidence interval of 0.95, the Gibbs energy (ΔG_β_) was determined from Equation (10) by linear regression. According to Alpizar-Reyes et al. (75), positive values of Gibbs energy (+ΔG_β_) are an indicative criterion that sorption is non-spontaneous, while negative values (–ΔG_β_) provide information on the spontaneity of sorption. Thus, the values of ΔG_β_ present in Table 7 suggest that the sorption process was non-spontaneity for all the samples. In addition, by analyzing the Gibbs energy data (ΔG_β_) reported in Table 7, a significant effect of gamma irradiation remarkably appears. Indeed, the control samples presented lower free energy (ΔG_β_) compared to the irradiated samples. In addition, as the irradiation dose increases, the free energy decreases. Whereas, high Gibbs free energy values indicate high availability of water adsorption and high hydrophilic properties, the study of thermodynamic properties for agro-products such as the free energy (ΔG_β_) is of major importance since it generally generates new data on the association and energy between water molecules in the system (76).

**Table 7:** Gibbs energy (ΔG_β_) for control and gamma irradiated (1, 1.5, and 2 kGy) dried figs.

**Samples**	**ΔG_β_ (J.mol^−1^)**	***T*_β_ (K)**
Control	137.1	326.41
1 kGy	498.4	310.59
1.5 kGy	277	300.45
2 kGy	259.4	297.12

### Enthalpy–entropy compensation theory

A simple thermodynamic argument suggests that the enthalpy–entropy compensation is a general property of weak intermolecular interactions and that the two free energy contributions should almost equilibrate for hydrogen bonding at 300 K. This statement from Dunitz (77) also drive him to ask how can we estimate the contributions of entropy and enthalpy to the free energy of association at varying binding forces. Thus, according to Liu and Guo (78), if there is a linear correlation between the logarithm of pre-exponential factors and activation energies, between enthalpies and activation entropies, or between changes in enthalpy and entropy of a series of similar reactions, the compensation effect is true. However, this definition is proposed based on the assumption that the data used in the correlation is error-free. Nevertheless, in real experiments, measurement errors are inevitable and the data used in the correlation are the estimators of the corresponding parameters. Therefore, it is possible that although the true values of the parameters do not correlate, their estimators do, and this is the cause of false compensation.

**Figure 7:**
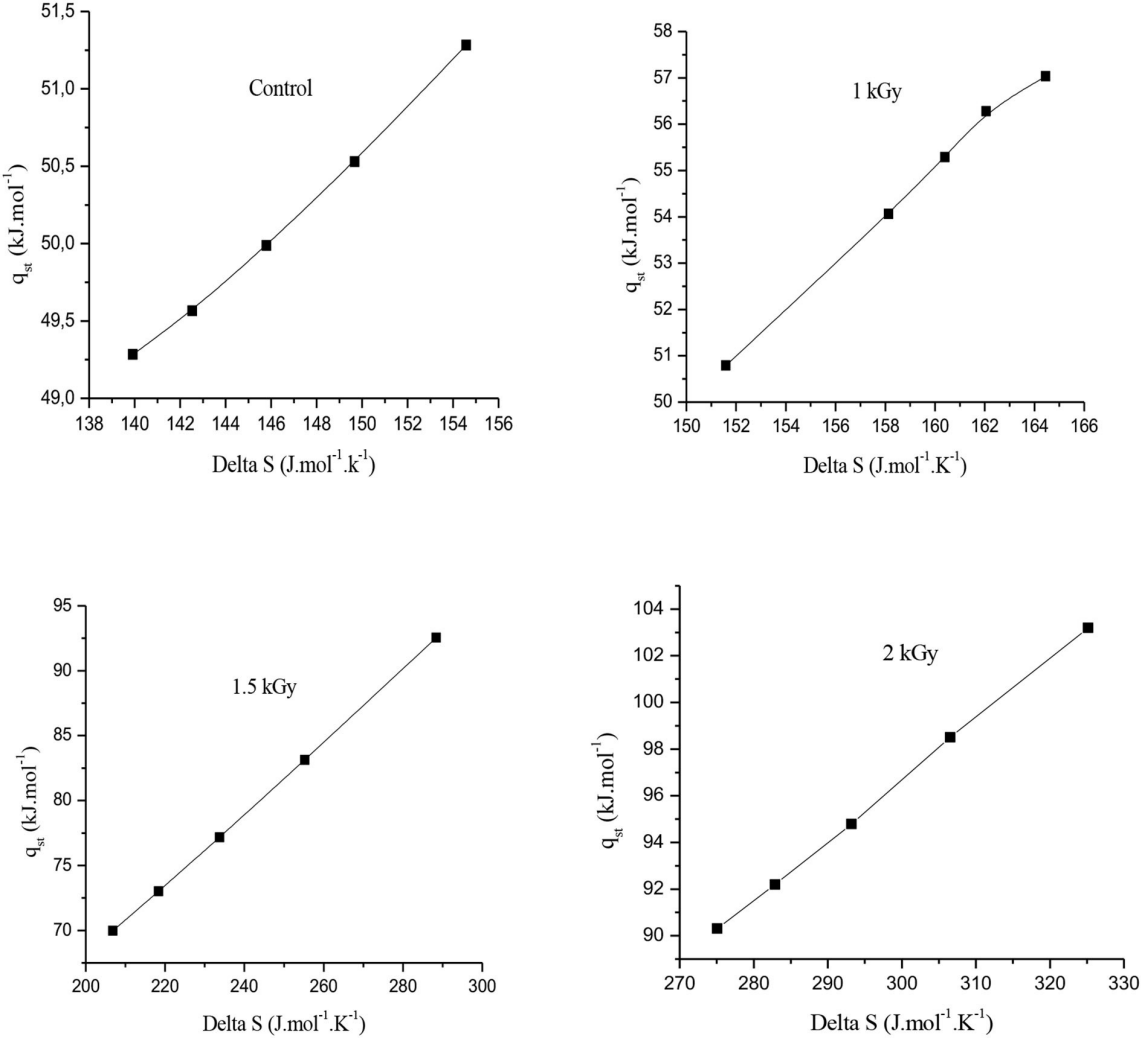
Enthalpy–entropy compensation theory for the water adsorption processes.

Figure 7 illustrates the graphs of net isosteric heat of sorption (q_st_) vs. differential entropy (ΔS) for each sample category, control and irradiated. A high degree of linearity between (*q*_*st*_) and (ΔS) is observed, which assumes that the theory of compensation exists (38, 74). By examining the illustrations in Figure 7, the realization of the compensation theory implies that a single reaction mechanism is followed by all members of the series of reactions; therefore, the existence of a single mechanism suggests that the microstructure of food is stable and does not undergo any change during moisture sorption (69, 79, 80). This observation joins that of Beristain et al. (81), concluding that the linear plot of compensation between *q*_*st*_ and ΔS correlates well with water adsorption in starchy and sugar-rich foods, such as dried figs. In addition, with our results, it can be assumed that the gamma irradiation treatment did not modify this compensation. With regard to the compensation enthalpy–entropy theory, the isokinetic temperature (T_β_) and the harmonic temperature (T_hm_) are significantly, at a certain distance, different from one another. The calculated harmonic temperature (T_hm_) was 312.94 K, while the observed isokinetic temperature values were 310.59 K, 300.45 K, and 297.12 K for 1, 1.5, and 2 kGy, respectively. This difference supports the enthalpy–entropy compensation theory. Therefore, data, presented in this study, showed that (T_hm_) is higher than (T_β_), suggesting that the adsorption process of dried fig samples is entropy-driven (35, 38, 82, 83).

## Conclusion

Stability of dried foods, as easily accessible snacks, continues to be a major concern for the agri-food industry. Therefore, studying the effect of gamma irradiation, as being a non-heating and non-toxic processing technology, and environmentally friendly, on dried food adsorption isotherms can be very useful to particularly assess it could probably modify the metabolism and the water retention capacity and potentially water activity of the matrix. In this study, the thermodynamic properties derived from dried figs' moisture adsorption isotherms provided relevant information about the influence of irradiation at increasing doses on samples' hygroscopic properties under different storage conditions. The magnitude of ^60^Co γ-rays effect becomes more important as the irradiation dose increases. At the end of this study, gamma irradiation optimized the maximum temperature thresholds and the final water contents used for storage to ensure the stability of the dried figs during storage. As the application of gamma irradiation in the food industry is generally framed on each side by minimum value allowing the desired objective to be achieved, and a maximum value depending on the cost of treatment, and on the other hand, the product's tolerance to radiation, this study, which remains the first, suggests the use of gamma rays at a dose up to 2 kGy as a promising approach to extend the shelf life of dried figs in terms of food stability linked to water activity.

## Data availability statement

The original contributions presented in the study are included in the article/supplementary material, further inquiries can be directed to the corresponding author/s.

## Author contributions

LH and AI conceived and performed the project, designed, and managed the experiments. LH, AI, and RO performed the experiments and data analysis, collected data, interpreted and discussed the data, and drafted the manuscript. LH and RO contributed to the revision of manuscript. RR contributed to the data curation and visualization. MM performed irradiation experiments. All authors approved the submission of the manuscript for publication.

## Conflict of interest

The authors declare that the research was conducted in the absence of any commercial or financial relationships that could be construed as a potential conflict of interest.

## Publisher's note

All claims expressed in this article are solely those of the authors and do not necessarily represent those of their affiliated organizations, or those of the publisher, the editors and the reviewers. Any product that may be evaluated in this article, or claim that may be made by its manufacturer, is not guaranteed or endorsed by the publisher.
